# Editorial: Advances in emerging coronavirus identification and tracing methods

**DOI:** 10.3389/fmicb.2023.1200733

**Published:** 2023-04-28

**Authors:** Junping Peng, Yi-Wei Tang, Ziyong Sun

**Affiliations:** ^1^National Health Commission (NHC) Key Laboratory of Systems Biology of Pathogens, Institute of Pathogen Biology, Chinese Academy of Medical Sciences & Peking Union Medical College, Beijing, China; ^2^Key Laboratory of Respiratory Disease Pathogenomics, Chinese Academy of Medical Sciences and Peking Union Medical College, Beijing, China; ^3^Department of Medical Affairs, Danaher Diagnostic Platform/Cepheid (China), New York, NY, United States; ^4^Department of Clinical Laboratory, Tongji Hospital, Tongji Medical College, Huazhong University of Science and Technology, Wuhan, China

**Keywords:** coronavirus, identification, nucleic acid amplification testing, antigen, antibody, tracing

The severe acute respiratory syndrome coronavirus 2 (SARS-CoV-2) gave rise to a viral pneumonia outbreak in China late 2019 and has continued spread rapidly across the globe becoming an unprecedented pandemic for over 3 years (Lu et al., 2020; Rambaut et al., 2020; Zhu et al., 2020). The World Health Organization (WHO) has reported more than 600 million confirmed coronavirus disease 2019 (COVID-19) cases and over six million deaths globally (https://covid19.who.int). Soon after the first COVID-19 case was reported and spread, the SARS-CoV-2 virus genome mutations gave rise to new variants, five of which have been classified as variants of concern (VOC) by the WHO (Alpha, Bata, Gamma, Delta, and Omicron), which also spread globally. SARS-CoV-2 VOCs can cause severe disease, increase infectivity, reduce neutralization by antibodies elicited from prior infections or vaccinations, and limit the efficacy of vaccine immunity (Karthikeyan et al., 2022; Oude Munnink and Koopmans, 2023). Therefore, rapid and convenient methods for detecting and tracing SARS-CoV-2 variants are required.

The Research Topic, “*Advances in emerging coronavirus identification and tracing methods*,” covered the latest developments and applications for differential diagnoses and origin tracing methods. The Research Topic contains 19 articles: five focused on monitoring the emergence of variants, 10 emphasized the development, evaluation, comparison, and application of efficient and easy-to-use methods (the targets of which are usually nucleic acids, antigens, and antibodies), and four expanded the application of mechanistic research.

The increasing prevalence of new SARS-CoV-2 variants can alter viral transmissibility, virulence, antigenicity, and recognition by the adaptive immune system triggered by prior vaccination; thus, characterizing and cataloging viral variants are crucial. Five articles were included related to this theme. First, Padilla-Blanco et al. performed a pilot study exploring viral variant circulation on Sicily Island, for which variation information is scarce. This study used reverse transcription polymerase chain reaction (RT-PCR) amplification and sequencing of selected viral genomic regions to characterize the variants, providing vital information on the predominant variants and their circulation during a specific pandemic wave in this insular region. Moreover, the SARS-CoV-2 virus evolves from mutations and the natural selection of variants. To address this issue, Tsuchiya et al. performed a comprehensive genomic analysis of 112 SARS-CoV-2 strains detected in Japan and simultaneously processed the genomic data from all of Japan deposited in the Global Initiative on Sharing Avian Influenza Data to investigate the pattern of mutations and examine the relationship between amino acid changes and the transmissibility and severity of each strain or lineage. However, as stated in the article, the lack of experimental evidence employing recombinant SARS-CoV-2 with or without particular amino acid alterations to support the impact of mutations was a drawback of this study. Similarly, to understand the molecular determinants associated with mutation-driven evolution, Pal et al. adapted nanopore sequencing to investigate the molecular evolution of the SASR-CoV-2 genome during the first, second, and third COVID-19 waves in Uttar Pradesh, India, which helped identify the key mutational combinations that led to vaccination failures in India and shed light on how the virus's binding affinity changed. Moreover, to explore SARS-CoV-2 evolution in specific populations, Hosaka et al. targeted immunosuppressed patients in a nosocomial cluster in Japan, performing whole-genome sequencing analyses to examine the evolution of mutations in this cluster. As a result, they identified evidence of emerging mutation acquisition during transmission, emphasizing the necessity of improved infection control measures to prevent nosocomial infections among immunosuppressed patients. Finally, SARS-CoV-2 shedding via human feces has resulted in viral genome detection in human sewage. Therefore, Barbé et al. developed a wastewater-based sequencing process to track shifts in variant predominance using Oxford Nanopore Technology, which is time-efficient and cost-saving. Notably, constrained by the complexity of SARS-CoV-2 strains in the sewage matrix, this approach could only detect mutations in conjunction with a genome site instead of strains, which is indirect proof of the presence of a lineage. Together, these articles highlight the urgent need to develop detection and tracing methods for SARS-CoV-2 with high sensitivity, specificity, and throughput.

Three articles were collected that compared detection and tracing methods and statistically analyzed SARS-CoV-2 variants. The need for tools to identify SARS-CoV-2-positive individuals is urgent. Thus, Cabrera et al. conducted a study early in the pandemic that assessed diagnostics methods for SARS-CoV-2 positivity. Wu et al. also assessed the impact of the COVID-19 pandemic on the population, finding that SARS-CoV-2 antibody assays may have an adjunct role in the diagnosis and exclusion of COVID-19, especially high-throughput technologies, such as enzyme immunoassays or chemiluminescent immunoassays (CLIAs). Finally, Windsor et al. compared multiple SARS-CoV-2 serology reference materials to the WHO International Standard (WHO IS) to determine their utility as secondary standards using an international network of laboratories with high-throughput quantitative serology assays. Their findings indicated that the arbitrary WHO IS unit does not accurately compare SARS-CoV-2 serology results between different laboratories or methods. This study also showed that even after converting to international or binding antibody units, candidate secondary material results differed drastically between the laboratory methods. Currently, there are three main SARS-CoV-2 detection methods: traditional culture, immunological, and molecular methods. Culture is the gold standard for pathogen identification. However, this method is technically demanding and time-consuming, thus, is not widely used for early SARS-CoV-2 screening. In contrast, molecular and immunological detection methods are common, simple, convenient, and rapid.

Additional seven research articles were included to analyze SARS-CoV-2 detection and tracing. First, Lin et al. developed an RAA/CRAA /CRISPR-Cas12a-mediated assay to specifically distinguish major SARS-CoV-2 variants. All reactions were conducted in sealed tubes without requiring complex equipments or facilities. Therefore, this simple and rapid assay could be implemented in resource-limited settings. Furthermore, these methods can be simplified for high-throughput multiplex screening in combination with sophisticated microfluidic devices. Yu et al. developed a visual nucleic acid detection method combining reverse transcription loop-mediated isothermal amplification and a vertical flow visualization strip (i.e., RT-LAMPVF), which does not require special equipment, has broad applicability, and is expected to achieve on-site real-time detection without needing to transport samples, making it especially useful for screening in airports and train stations. Tanimoto et al. quantified the SARS-CoV-2 RNA copy number using reverse transcription quantitative real-time PCR with primers and probes targeting the N gene, allowing for the detection of both wild-type and variant SARS-CoV-2 strains in sewage samples from two wastewater treatment plants in Kobe City, Japan, during the fourth and fifth COVID-19 pandemic waves (between February 2021 and October 2021). They found that quantitative RNA studies in sewage could be useful for administrative purposes related to public health, including issuing warnings and implementing preventive measures within sewage basins. Regarding immunological detection methods, Wang et al. obtained two monoclonal antibodies that recognized the recombinant porcine Delta coronavirus nucleocapsid protein, reporting high coincidence rates compared to reverse transcription-quantitative PCR results in only 15 min, allowing for rapid diagnosis and early control of the disease. Moreover, Choi et al. compared three CLIAs, three lateral flow immunoassays, and a surrogate virus neutralization test assay. With vaccine administration, routine antibody tests for COVID-19 were also initiated in general laboratories worldwide. Therefore, to select the most suitable serological assay for a particular laboratory environment, it is necessary to understand the characteristics of each assay. Additionally, Cai et al. evaluated non-specific reactivity in SARS-CoV-2 serological tests in 46,777 post-pandemic samples, reporting an unspecific reactivity incidence rate of 0.361% involving 14 disease spectrum categories. These results indicate that unspecific reactivity must be excluded when using serological antibody testing for COVID-19 epidemiological analysis or virus tracing. In addition, nanopore sequencing is increasingly used for whole- genome sequencing of SARS-CoV-2 since it is simple, fast, and provides real-time results. Finally, Luo et al. found that the Q20+ kit was more accurate than previous nanopore sequencing kits, especially for sequencing long amplicons, which could promote epidemic prevention and control and improve SARS-CoV-2 traceability analyses. An essential component, such as proteins or lipids, may influence viral replication and interaction activities. Therefore, several mechanistic studies have explored these correlations, which could help expand treatment strategies.

To clarify whether non-structural proteins (nsps) are indispensable for viral replication and transcription, Jin et al. examined the replication activity of the viral replicon by deleting individuals nsps. They discovered that the dependence of viral replication on individual nsps varied significantly, providing a new perspective on the role of nsps in viral replication and transcription. This information also suggests that nsps are a potential target for antiviral drug development. Strategies or approaches that could lead to therapeutic options for SARS-CoV-2 are also of interest. For example, Wang et al. utilized phage display to search for peptides that likely inhibited S protein binding to cellular angiotensin-converting enzyme 2 (ACE2). As a result, they identified two potential 12-aa peptides, which were further exploited to produce peptidomimetics, the intercepts of which were verified experimentally. It is worth emphasizing that these peptides or their derivatives may be developed into therapeutic regimens that interrupt virus-host attachment and hinder disease onset. Furthermore, Ishigaki et al. investigated the protective efficacy of the rDIs-S vaccine, a recombinant DI strain carrying the SARS-CoV-2 spike-encoding gene, against SARS-CoV-2 infection in a non-human primate model and heterologous human ACE2-expressing mouse model. The results indicated that vaccination with rDIs-S could prevent protein expression related to the severe pathogenic effects of SARS-CoV-2 infection and restore protein expression related to immune responses. Moreover, previous studies have reported dyslipidemia in patients with COVID-19, thus, Zhao et al. conducted an extensive study comparing serum lipid profiles among different cohorts and a bioinformatics analysis to explore the possible relationship between viral pathogenesis and metabolic reprogramming mechanisms, which may be a target for developing antivirals against SARS-CoV-2.

This review topic comprised studies on advances in coronavirus identification and tracking methods, emphasizing efficient and easy-to-use methods. These methods target nucleic acids, antigens, and antibodies and apply to coronavirus identification, differential diagnoses, and origin tracing. Collectively, these studies considerably benefit the disease research field by presenting ways to cut off transmission routes and formulating epidemic prevention and control strategies for COVID-19.

## Author contributions

JP was a guest associate editor of the Research Topic and wrote the paper text. Y-WT and ZS were guest associate editors of the Research Topic and edited the text, respectively. All authors contributed to the article and approved the submitted version.
